# Educational Interventions in Adults with Type 2 Diabetes Mellitus in Primary Health Care Settings. A Scoping Review*

**DOI:** 10.17533/udea.iee.v41n2e15.

**Published:** 2023-08-29

**Authors:** Yasmin Alejandra Castillo-Merino, Camila Ospina-Ayala, Natalia Esquivel-Garzón, Alba Luz Rodríguez-Acelas, Wilson Cañon-Montañez

**Affiliations:** 1 RN, M.Sc, Ph.D candidate. Faculty of Nursing, Universidad de Antioquia. Medellín, Colombia. Email: yasmin.castillo@udea.edu.co https://orcid.org/0000-0002-1442-1725 Universidad de Antioquia Faculty of Nursing Universidad de Antioquia Medellín Colombia yasmin.castillo@udea.edu.co; 2 RN, M.Sc, Ph.D candidate. School of Medicine, Pontifícia Universidade Católica do Rio Grande do Sul. Porto Alegre - RS, Brazil. Email: camila.ayala@edu.pucrs.br https://orcid.org/0000-0003-1414-7495 Pontifícia Universidade Católica do Rio Grande do Sul School of Medicine Pontifícia Universidade Católica do Rio Grande do Sul Porto Alegre RS Brazil camila.ayala@edu.pucrs.br https://orcid.org/0000-0003-1414-7495; 3 RN, Ph.D, Assistant Professor. School of Nursing, Universidad Industrial de Santander. Bucaramanga, Colombia. Email: nesquiva@uis.edu.co https://orcid.org/0000-0002-5354-6774 Universidad Industrial de Santander School of Nursing Universidad Industrial de Santander Bucaramanga Colombia nesquiva@uis.edu.co https://orcid.org/0000-0002-5354-6774; 4 RN, M.Sc, Ph.D, Associate Professor. Faculty of Nursing, Universidad de Antioquia. Medellín, Colombia. Email: aluz.rodriguez@udea.edu.co https://orcid.org/0000-0002-7384-3522 Universidad de Antioquia Faculty of Nursing Universidad de Antioquia Medellín Colombia aluz.rodriguez@udea.edu.co; 5 RN, M.Sc, Ph.D, Associate Professor. Faculty of Nursing, Universidad de Antioquia. Medellín, Colombia. Email: wilson.canon@udea.edu.co. Corresponding author. https://orcid.org/0000-0003-0729-5342 Universidad de Antioquia Faculty of Nursing Universidad de Antioquia Medellín Colombia wilson.canon@udea.edu.co

**Keywords:** diabetes mellitus, type 2, primary health care, patient education as topic, self-care, primary care nursing, diabetes mellitus, tipo 2, atención primaria en salud, educación del paciente como asunto, autocuidado, enfermería de atención primaria, diabetes mellitus, tipo 2, atenção primária em saúde, educação do paciente como assunto, autocuidado, enfermagem de atenção primária

## Abstract

**Objective:**

To synthesize the evidence of studies with educational interventions for adults with type-2 diabetes mellitus (DM2) in primary health care settings**.**

**Methods:**

A scoping review was conducted following the recommendations by the Joanna Briggs Institute and by the PRISMA declaration. The protocol was registered in INPLASY20215009. The search was carried out in: MEDLINE (via PubMed), EMBASE, Web of Science, LILACS, and grey literature.

**Results:**

Seventeen studies were included; most were randomized clinical trials of which 65% were conducted in high-income countries, and all the studies represented 5 656 participants. The results showed four big categories derived from educational interventions: therapeutic adherence (significant results on the satisfaction with the treatment); self-care and self-management in diabetes (improvement in self-efficacy, empowerment, and disease awareness); glycemic control in diabetes (significant results in reducing glycosylated hemoglobin); nursing and its role in the educational interventions on patients with DM2 (guidance in restructuring behaviors).

**Conclusion:**

The findings of this review suggest that educational interventions on patients with DM2 within the setting of primary health care can impact positively on therapeutic adherence, self-control, and knowledge of the disease. Moreover, it was possible to identify the influence of multidisciplinary health teams, where the relevance of nursing professionals in the construction and implementation of educational interventions is evidenced in obtaining better health results.

## Resumen

**Objetivo:**

Sintetizar la evidencia de estudios con intervenciones educativas para adultos con diabetes mellitus tipo 2 (DM2) en la atención primaria de salud.

**Métodos:**

Se realizó una revisión de alcance siguiendo las recomendaciones del *Instituto Joanna Briggs* y de la declaración PRISMA. El protocolo se registró en INPLASY20215009. La búsqueda se realizó en: MEDLINE (vía PubMed), EMBASE, Web of Science, LILACS y literatura gris.

**Resultados:**

Diecisiete estudios fueron incluidos, la mayoría fueron ensayos clínicos aleatorizados, de estos 65% fueron conducidos en países de ingresos altos, y todos los estudios en total representaron 5656 participantes. Los resultados mostraron cuatro grandes categorías derivadas de las intervenciones educativas: adherencia terapéutica (resultados significativos en la satisfacción con el tratamiento); autocuidado y automanejo en diabetes (mejora en la autoeficacia, empoderamiento y conciencia de la enfermedad); control glucémico en diabetes (resultados significativos en la reducción de la hemoglobina glicosilada); enfermería y su papel en las intervenciones educativas en pacientes con DM2 (orientación en la reestructuración de comportamientos).

**Conclusión:**

Los hallazgos de esta revisión sugieren que las intervenciones educativas en pacientes con DM2 en el ámbito de la atención primaria de salud pueden impactar positivamente en la adherencia terapéutica, el autocontrol y el conocimiento de la enfermedad. Además, fue posible identificar la influencia de los equipos multidisciplinarios de salud, donde se evidencia la relevancia de los profesionales de enfermería en la construcción e implementación de intervenciones educativas para la obtención de mejores resultados de salud.

## Resumo

**Objetivo:**

Sintetizar as evidências de estudos sobre intervenções educacionais para adultos com diabetes mellitus tipo 2 (DM2) na atenção primária à saúde.

**Métodos:**

Foi realizada uma revisão de escopo seguindo as recomendações do Joanna Briggs Institute e a declaração PRISMA. O protocolo foi registrado no INPLASY20215009. A pesquisa foi realizada em: MEDLINE (via PubMed), EMBASE, Web of Science, LILACS e literatura cinzenta.

**Resultados:**

Dezessete estudos foram incluídos, a maioria eram ensaios clínicos randomizados, 65% deles foram conduzidos em países de alta renda e todos os estudos, no total, contaram com 5656 participantes. Os resultados mostraram quatro grandes categorias derivadas das intervenções educacionais: adesão (resultados significativos na satisfação com o tratamento); autocuidado e autogestão da diabetes (melhoria na autoeficácia, fortalecimento e conscientização sobre a doença); controle glicêmico na diabetes (resultados significativos na redução da hemoglobina glicada); enfermagem e seu papel nas intervenções educacionais em pacientes com DM2 (orientação na reestruturação de comportamentos).

**Conclusão:**

Os achados desta revisão sugerem que as intervenções educacionais em pacientes com DM2 no ambiente da atenção primária à saúde podem impactar positivamente na adesão, no autogerenciamento e no conhecimento da doença. Além disso, foi possível identificar a influência das equipes multidisciplinares de saúde, onde fica evidente a relevância dos profissionais de enfermagem na construção e implementação de intervenções educacionais para obter melhores resultados de saúde.

## Introduction

Diabetes mellitus type 2 (DM2) is one of the non-communicable diseases that make up the high burden of morbidity and mortality in the world, representing a considerable public health problem.^1^ In agreement with International Diabetes Federation, there are 463 million adults with diabetes worldwide, and it is estimated that this number will increase to 578 million by 2030 and 700 million by 2045, since DM2 represents 90% of cases in the world and among people aged 50 to 74 years it is the fifth cause of death.^2^ Different strategies have been used in health services since the performance of the interdisciplinary team, seeking to face a problem that is expanding globally.^3^ However, for this, the commitment that the patient assumes with his care is decisive.

The programs conducted for self-care in patients with DM2 have been widely addressed by different studies that have been able to identify the benefits of the implementation of educational interventions in the context of primary health care for patients with DM2. These benefits are specifically identified in activities that promote a healthy lifestyle, motivating self-efficacy and a better level of adherence and disease control.^4^ In the development of programs focused on the individual, in order to contribute to decision-making and the search for a change in some patterns of risk in lifestyle, strategies that impact on primary health care services are seen as relevant.^4^ The literature shows that the use of educational interventions in patients with DM2, compared to habitual care, can improve self-control and the management of clinical parameters as well as reduce costs in health systems.^5^ Therefore, educational interventions have a high level of importance within self-care. Consequently, seeking to condense the literature, discover new strategies framed in the interventions, and call the attention of nursing professionals to the urgent need to deal with this problem, this scoping review aimed to synthesize the evidence on educational interventions for DM2 in primary health care.

## Methods

Design and registration of the protocol. This is a scoping review (SR) guided by the recommendations of the Joanna Briggs Institute (JBI);^6^ and followed the Preferred Reporting Items for Systematic Reviews and the Meta-Analyses (PRISMA) checklist for scoping reviews.^7^ The protocol was registered under the serial number INPLASY202150091.^8^

Source of data and search strategy. Searches were performed in the following databases: MEDLINE (via PubMed), Excerpta Medica Database (EMBASE), Latin American Caribbean Health Sciences Literature (LILACS via BIREME), and Web of Science. In addition, gray literature was considered in the selection process. These searches were performance out from inception until March 2021 as indicated in Supplemental online 1.The following search strategy was used for MEDLINE: *(Diabetes Mellitus, Type 2[MeSH Terms]) OR (Diabetes Mellitus, Type II) OR (Diabetes, Type 2) OR (Type 2 Diabetes) OR (Type 2 Diabetes Mellitus) AND (primary health care[MeSH Terms]) OR (Care, Primary Health) OR (Health Care, Primary) OR (Primary Healthcare) OR (Healthcare, Primary) AND (Education[MeSH Terms]) OR (Patient education[MeSH Terms]) OR (Education, Patient) OR (Patient Education) OR (Education of Patients) OR ((Health Education[MeSH Terms]) OR (Education, Health) OR (Education, Nursing[MeSH Terms]) OR (Nursing Education) OR (Educations, Nursing) OR (Nursing Educations) AND (Standard of Care[MeSH Terms]) OR (Care Standard) OR (Care Standards) OR (Standards of Care).*

Eligibility criteria of the studies. This SR includes randomized controlled trials (RCTs), quasi-experimental, and cluster studies, published from inception until March 2021 in the languages of Portuguese, English, or Spanish, with both the abstract and full text available. The following PICO strategy (population, intervention, comparator, outcomes) was applied for study eligibility, P: adults with DM2 in primary health care, I: educational interventions; C: habitual or standard care, and O: improve clinical outcomes (adherence or compliance to treatment, diabetes control, knowledge and self-care). 

Data extraction. The extracted data was collected in an Excel spreadsheet, containing the following information: author, year, country, study design, sample size, type of intervention, follow-up, control group, and main findings. 

Risk of bias assessment. The risk of bias tool (RoB 1) from the Cochrane Collaboration was used to evaluate the risk of bias in RCTs. The following elements were evaluated: random sequence generation and allocation concealment, blinding of participants and personnel, blinding of outcome assessment, incomplete outcome data, and selective reporting of results and other sources of bias.^9^ In addition, the JBI recommendations were used to assess the level of evidence of the studies.^10^ For the graphical visualization of the result of the methodological quality of the individual studies, it was carried out in the *robvis* web application. ^11^

## Results

### Identification and selection of the studies

**Figure 1:**
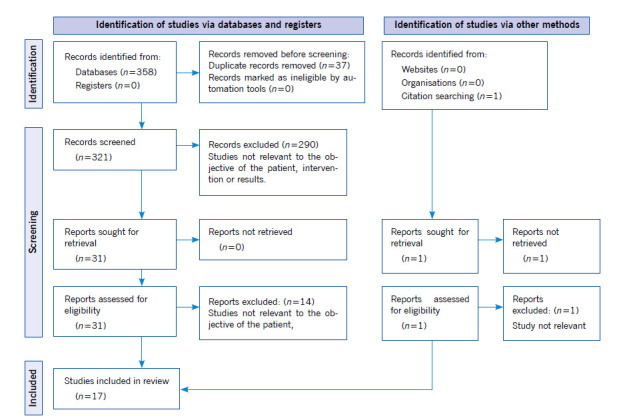
Flow diagram of study selection process

In total, 358 studies were identified (Figure 1). Of these, 37 duplicate articles were excluded, 321 studies being included for reading the title and abstract. Of those 321, 290 studies were excluded because they did not meet the objectives of the type of patient, type of study, educational intervention, location, or results found. Of the 31 remaining studies included for full text reading, 14 articles that did not meet the criteria established in the PICO strategy were excluded. Finally, seventeen studies meet the eligibility criteria for inclusion in this scoping review.

### Characteristics of studies included

Of the 17 studies included in the SR. The studies were carried in 11 countries; of these, 65% of the studies were conducted in high-income countries, the rest being from low- and middle-income countries. These studies were published between 2010 and 2020. In relation to the type of study, it was found that 13 of the studies were randomized controlled trials and four cluster-randomized trials. Sample sizes ranged from 76 to 1589, with a mean of 344. The included studies were followed up for different periods, ranging from the first month after the intervention to 24 months. Studies with follow-up at 3, 6, and 12 months predominated. Although educational interventions showed a wide diversity, common strategies were found, such as education for diabetes control, a diabetes adherence and empowerment program, activities that included the individual, family, and communities. Education focused on the knowledge of the disease, warning signs, diet, and self-care practices. Regarding the control group, it was evident that all studies included standard care, that is, usual care. Educational interventions for the management of adults with DM2 were identified, which were grouped into four large categories, with the goal of projecting a better understanding of this review and a more delimited guide that serves health professionals in the implementation of interventions that respond to the needs identified in their practical environments.

### Therapeutic adherence

Therapeutic adherence has historically represented one of the most important elements in the care of patients with DM2 and, with it, the execution of adequate treatment and control of the disease. Therefore, different countries have focused their educational intervention programs on adequate therapeutic adherence and, with this, seek to contribute to the management of DM2.^12^ Thus, as a study aimed to provide training to community health workers on the pathophysiology of the disease, risk factors for DM2, and lifestyle strategies with an impact on glycemic control (nutrition, exercise, physical activity, and prevention of diabetic complications), this educational intervention, which was received by the professionals who guided the patients with DM2, allowed a greater adherence to the treatment, which consequently brought the improvement of the blood glucose levels of the study participants.

**Table 1:** Characteristics of the included studies

Authors, (year), country	Type of study	Sample size	Intervention group (IG)	Follow-up	Control group (CG)	Main findings	Level of evidence
Chen *et al.* (2020)^13^ China	Randomized clinical trial	1325	Education conferences	12 months	Standard care	Blood glucose level decreased in the IG compared to the CG:	1C
		Intervention: 665	Periodic follow-up interviews with physical examination			Difference-in-difference model (DID) = 0.53mmol (95% CI 0.90, to 0.16); *p*=0.005	
		Control: 660	Specialized medical services			Diabetes knowledge score increased significantly in the IG compared to CG:	
						DID = 0.91 (95 % CI 0.64-1.18)	
De la Fuente *et al.* (2020)^14^ Spain	Randomized controlled clinical trial	236	Structured education provided by a nurse:	12 and 24 months	Standard care	Glycated hemoglobin (HbA1C)	1C
		Intervention: 97	Accompaniment to a family member or caregiver			IG:(−0.55, 95% CI −0.20, −0.90; *p*<0.001)	
		Control: 139	Basic knowledge of diabetes			CG: (0.06, 95% CI −0.14, +0.28, *p*=0.530)	
			Use of empowerment model			HbA1C <7%:	
						IG: 35.2% vs. CG: 24.7%	
Presley *et al.* (2020)^15^ United States	Randomized controlled trial	97	Community-based diabetes self-management education and peer support through the mHealth web application:	6 months	Standard care	HbA1C reduction after 6 months:	1C
		Intervention: 62	12 weekly phone calls			IG: 10.1 (SD 1.7) to 9.6 (SD 1.9)	
		Control: 35	3 monthly calls			CG: 9.8 (SD 7) to 9.1 (SD 1.9)	
						Reduction of diabetes distress in both groups:	
						*p*<0.001	
White *et al*. (2020)^16^ United States	Cluster randomized clinical trial	364	Partnership to improve diabetes education:	12 and 24 months	Standard care	Treatment effects on 12 months:	1C
		Intervention: 184	Literacy-sensitive, provider-centered health communication intervention			Adjusted HbA1C:	
		Control: 180				IG: (−0.76 [95% CI, −1.08 to - 0.44]; *p*<0.001	
						CG: (-0.54 [95% CI, - 0.86 to - 0.21]; *p=*0.001)	
						Satisfaction with treatment:	
						IG: (3.93 [95% CI, 2.48-6.21]; *p*<0.001)	
						CG: (3.04 [95% CI, 1.93-4.77]; *p*<0.001)	
						Self-efficacy:	
						IG: (2.97 [95% CI, 1.89-4.67]; *p*<0.001)	
						CG: (1.81 [95% CI, 1.1-2.84]; *p*=0.01)	
Sharoni *et al*. (2018)^17^ Malaysia	Randomized controlled trial	76	Health education program, based on:	1 month and 3 months	Standard care	Foot self-care behavior after 12 months:	1C
		Intervention: 36	Theory of self-efficacy of Albert Bandura			IG: 62.61 (SD 7.54)	
		Control: 36	Group Diabetes Education Seminars			CG: 47.55 (SD 7.30)	
						Foot care self-efficacy after 12 months:	
						IG: 40.89 (SD 4.91)	
						CG: 34.37 (SD 4.69) Knowledge of foot care after 12 months:	
						IG: 7.68 (SD 1.49)	
						CG: 5.16 (SD 3.09)	
Santos *et al*. (2017)^18^ Brazil	Cluster randomized clinical trial	238	Adherence and empowerment program in diabetes:	12 months (3-months intervals)	Standard care	Glycemic control (HbA1c):	1C
		Intervention: 127	Group education			IG: 7.10 (5-12.4); *p*=0.001	
		Control: 111	Home visits			CG: 7.40 (4.9-13.9); *p*=0.3000	
			Telephone monitoring			Self-care questionnaire for DM2:	
						IG: 4.05 (1.75-6.25); *p*=0.0001	
						CG: 3.00 (1.25-6.1); *p*=0.9700	
						Empowerment questionnaire for DM2:	
						IG: 4.13 (2.75-5); *p*=0.001	
						CG: 4.00 (2.5-4.88); *p*=0.001	
Paz-Pacheco *et al*. (2017)^19^ Filipinas	Randomized clinical trial	155	Diabetes self-management education:	3 and 6 months	Standard care	Glycemic control (HbA1C≤7.0) after 6 months: n (%)	1C
		Intervention: 85	During the follow-up visits, 8 topics were taught.			IG: 43 (59.72)	
		Control: 70				CG: 20 (38.46)	
						Foot examination after 3 months: n (%)	
						IG: 49 (76.56) vs. CG: 34 (57.63)	
Grillo *et al.* (2016)^20^ Brazil	Randomized clinical trial	137	Diabetes self-management education:	12 months	Standard care	Knowledge of diabetes mellitus after 12 months:	1C
		Intervention: 69	Identification of modifiable factors			IG: 16 (3%)	
		Control: 68	Non-pharmacological treatment			CG: 12 (4%)	
			Drug therapy			Glycemic control (HbA1C) after 12 months:	
			Complications of chronic diabetes			IG: 8.7 (1.7%) vs. CG: 9.2 (2.2%)	
			Foot care				
Pérez-Escamilla *et al*. (2015)^12^ United States	Randomized clinical trial	211	Latino Diabetes Best Practices Program:	3, 6, 12 and 18 months	Standard care	Glycemic control (HbA1C) after 18 months:	1C
		Intervention: 105	Self-management of diabetes			IC: 9.32 (8.91, 9.74)	
		Control: 106	Medications for diabetes			CG: 8.77 (8.35, 9.20)	
			Nutrition and exercise				
			Intercultural counseling				
			Mental health				
Merakou *et al.* (2015)^21^ Grecia	Clinically controlled trial	193	Structured group educational program:	Not informed	Standard care	Glycemic control (HbA1C) after 6 months:	1C
		Intervention: 138	Conversation Maps: Learning About Diabetes			IG: 1.4 (95% CI: 1.1, 1.7; *p*<0.001)	
		Control: 55				CG: 0.5 (95% CI: 0.5, 0.3; *p*=0.003)	
						Maps for people with DM2 are more effective in diabetes self-management	
Ruggiero *et al*. (2014)^22^ United States	Randomized controlled clinical trial	266	Physician Assistant Self-Care Coaching:	6 and 12 months	Standard care	Medication adherence:	1C
		Intervention: 134	Patient-centered and individualized			IG: 6.6 (SD 2.0)	
		Control: 132	Transtheoretical model			CG: 6.12 (SD 2.4)	
			Empowerment model			Diabetes self-care behaviors:	
			Best practice advice			IG: 3.81 (SD 2.2)	
						CG: 3.48 (SD 2.2)	
						There results were not significant.	
Plotnikoff *et al*. (2011)^23^ Canada	Randomized clinical trial	96	Diabetes Education Program Plus Physical activity:	3, 6 and 12 months	Standard care	Glycemic control after 12 months:	1C
		Intervention: 49	Energy Expenditure and Fitness			IG: -0.5 (-0.9 to -0.2; *p*<0.01)	
		Control: 47	Modified Canadian Aerobic Fitness Test			CG: -0.4 (-0.7-0.0)	
			Phone support			Physical Activity after 12 months:	
						IG: 654.2 (466.9-841.6; (*p*<0.01)	
						CG: -33.9 (-213.6-145.8)	
Quinn *et al*. (2011)^24^ Canada	Cluster-randomized clinical trial	163	Mobile Diabetes Intervention:	12 months	Standard care	Glycemic control after 12 months:	1C
		Intervention: 107	Coach-only			IG: 1.9% (95% CI 1.5-2.3)	
		Control: 56	Coach primary care providers portal			CG: 0.7% (0.3-1.1)	
			Coach primary care providers portal with decision-support			There were no significant results in relation to diabetes distress, depression, diabetes symptoms, or blood pressure and lipid levels (all *p*>0.05).	
Sönnichsen *et al*. (2010)^25^ Austria	Cluster-randomized controlled trial	1489	Disease management programs “Therapie aktiv”:	12 months	Standard care	Glycemic control (HbA1C):	1C
		Intervention: 649	Group for Preventive Medicine Salzburg			IG: 0.41% [95 CI % 0.32; 0.50]	
		Control: 840	Standardized documentation of physical examination			CG: 0.28% [95 CI % 0.21; 0.35]	
			Structured interdisciplinary care			Eye examination:	
						IG: 71.0% vs. CG: 51.2%	
						Foot examination:	
						IG: 73.8% vs.CG: 45.1%	
						Patient education:	
						IG: 49.5% vs. CG: 20.1%	
Gaillard *et al*. (2015)^26^ United States	Randomized clinical trial	96	Diabetes Self-Management and Support:	6 months	Standard care	Glycemic control (HbA1C) after 6 months:	1C
		Intervention: 58	Community health worker			IG: 7.5 (1.3%; *p*=0.02)	
		Control: 38	Diabetes self-management training			CG: 7.7 (1.5%; *p*=0.405)	
			Weekly call support			No significant changes in metabolic parameters	
			Community resources				
Gehlawat *et al*. (2019)^27^ India	Randomized controlled trial	314	Diabetes Self-Care Activities:	6 months	Standard care	Self-care of the feet:	1C
		Intervention: 157	Education sessions of 45 minutes			IG: 3.64 vs. CG: 2.21	
		Control: 157	Self-care kits (mirror, an oil bottle, and glucose tablets)			Both groups: 1.95 (1.4-2.4; *p*<0.001)	
						Inspect the inside of your footwear:	
						IG: 1.34 vs. CG: 0.04	
						Both groups: 0.78 (0.5-1.0; *p*<0.001)	
Romero-Guevara *et al*. (2019)^28^ Colombia	Randomized controlled trial	200 Intervention: 98 Control: 102	Teaching: Individual:	6 and 12 months	Standard care	Systolic blood pressure in 24 (mmHg):	1C
			Six educational sessions of 20 to 40 minutes: Behavior modification; teaching, disease process, prescribed medication, prescribed diet and exercise and coping enhancement			IG: 125 (SD 14.6)	
			By two nurses			CG: 123 (SD 13.9)	
						HbA1c:	
						IG: 6.19 (SD 1.71)	
						CG: 6.15 (SD 1.44)	
						These results were not significant.	

In the United States, a study which carried two groups through an intervention using two guides on educational intervention for DM2 (one of the groups used the kit designed to improve diabetes education in the intervention, sessions were carried out that included updating on diabetes and instruction on techniques to improve communication in health, and the second group received guidance based on the National Health Program as an intervention. Diabetes Education to carry out discussions for the care of the disease) found that after these interventions in patients with diabetes, satisfaction with treatment presented significant results (3.93 [95% confidence interval (CI), 2.48-6.21]; *p*<0.001 versus 3.04 [95% CI, 1.93-4.77]; *p*<0.001), improving adherence to treatment.^16^ Another study through the delivery of material created by an interdisciplinary group based on the American Diabetes Association and the American Association of Diabetes Educators, was carried out with an educational intervention in small groups with 45-minute sections, focused on the self-care of “healthy eating, being active, regular blood sugar control, taking medication on time, problem solving, risk reduction and healthy coping,” resulting in 2% adherence to medication for control of blood glucose levels by the participants.^27^

### Self-care and self-management in diabetes

The evidence has shown the high morbidity rate that diabetes mellitus represents. In this sense, the implementation of educational interventions that are focused on the proper management of it will consequently allow a positive impact on the self-care of patients. Thus, a study that applied the theory of self-efficacy in the self-care behavior with the feet in adults with DM2, allowed to obtain improvements in the performance and indirect experience of the physical and emotional states and verbal persuasion of the participants.^17^The application of this theory has shown significant results in self-care and knowledge of DM2 when comparing the intervention group to which the theory was applied and the control group that received standard treatment (*p*<0.01).^16^ Structured education programs in primary health care settings have shown the effectiveness of self-care practices and a significant improvement of 33.5% [95% CI: 22.9-44.0].^27^ This, therefore, shows foot care’s considerable relevance since it seeks to also impact the patient's own self-care and thereby improve knowledge about the disease, which has led to significant results at 6 at 12 months of intervention (*p*<0.01).^22^ On the other hand, adherence and empowerment are indicators that can present improvement through self-care practices. A study carried out in Brazil implemented a strategy of group education and family visits. This group education strategy produced better results in relation to glycemic control and diabetes self-care.^18^ Through educational interventions, it has also been shown that knowledge about diabetes significantly increased in the group that received the educational intervention versus the control group (where it decreased), with the difference-in-difference model (DID) equal to (0.91 [95% CI: 0.64-1.18], *p*<0.001).^13^ Likewise, a study found that community-based, peer-supported education shows a significant reduction in diabetes distress (*p*<0.001).^15^

### Glycemic control of diabetes

The versatility of measures such as web applications for the education of patients with DM2 that have allowed a significant reduction in HbA1C (*p*=0.004).^15^ A study with the intervention with web portals showed a mean decrease in glycated hemoglobin of 1.9% in contrast to standard care 0.7%, which has a variance of 1.2% (*p*=0.001) at 12 months.^24^ The literature has been consistent in demonstrating the positive results of group programs for education in patients with DM2. Two studies showed significant results (*p*<0.001) in the reduction of glycated hemoglobin compared to other interventions, such as home visits, or standard care.^16^^,^^21^ Interventions in structured groups have also made it possible to improve the knowledge of patients in relation to DM2, and with this, they have prevented the elevation of HbA1C.^20^ The inclusion of cultural aspects in educational interventions in a Latino population residing in the United States achieved a significant reduction in the HbA1C difference at 3 months (*p*=0.043), followed by a reduction difference at 6 months (*p*=0.05) and finally at 18 months (*p*=0.009).^12^ Similarly, a culturally adapted self-care coaching intervention for racial/ethnic minority populations showed significant improvement in blood glucose levels.^22^ Likewise, the individualized educational intervention in a study carried out in Austria showed significant reductions in weight and cholesterol, but it did not significantly influence metabolic control measured by HbA1C after one year.^25^ With this, physical activity advice as an educational intervention has been effective in promoting a significant reduction in HbA1C 0.5% (*p*<0.01). Additionally, it has left positive results in glycemic control and the health of patients with DM2.^23^ These types of activities that provide accompaniment and support in lifestyle have shown that it is possible to obtain a significant reduction in HbA1C (*p*=0.02) and in random blood glucose levels (*p*=0.03), compared to standard care. Thus, approaching the patient as an integral being through empowerment and commitment undoubtedly allows for even more successful interventions for diabetes self-management.^19^^,^^26^

### Nursing and its role in educational interventions in patients with DM2

In the development of educational interventions, the multidisciplinary health team plays a fundamental role. However, it is recognized that nursing professionals have a differentiated scope within the team. Patient-centered interventions, which have the execution and accompaniment of the nursing staff, have allowed patients to self-identify their challenges and thus together be able to develop different strategies to overcome them.^26^ It has also been shown that the educational strategies that are stimulated by other educational components outside the standard, and that guide the restructuring of behaviors, through education on the disease process, prescribed medication, diet, prescribed exercise, and improvement in coping with the disease by nursing professionals in the care of patients with DM2, it has generated encouraging results.^28^ A study showed the importance of having professionals who have vast experience in education on DM2 through various structured and individualized educational interventions. The participants and their caregivers improved autonomy, allowing greater metabolic control and achievement of their long-term therapeutic goals.^14^ Educational interventions have shown a great role in the care of diseases. The evidence showed that the performance of the nursing professional in the execution of these interventions prevents the increase in HbA1C in patients with diabetes. This is possible through the training of groups with patients with DM2, through familiarization and training in diabetic education for the identification of risk factors, and the non-compliance with pharmacological treatment when compared to other educating agents.^20^

### Risk of bias of the studies included

**Figure 2:**
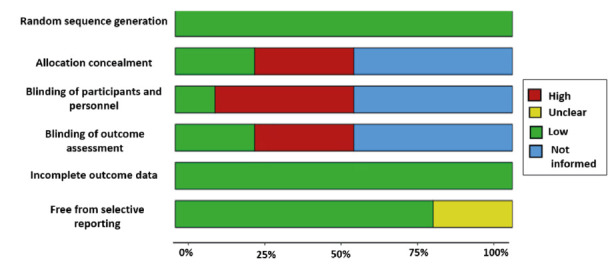
Methodological quality of individual studies

The results of the analysis of the quality of the included studies is presented in Table 2, performed based on the parameters evaluated by RoB 1 in the 17 included studies. 88% described adequate random sequence generation^12^^-^^16^^,^^18^^-^^20^^,^^22^^-^^28^ and 23% described allocation concealment.^17^^,^^24^^,^^25^^,^^28^ Only two articles described blinding of participants and staff,^12^^,^^28^ and 23% described blinding of outcome assessment.^12^^,^^14^^,^^20^^,^^28^ Regarding the risk of selective reporting of results, it was shown that 76% described the proposed results from the beginning^12^^-^^14^^,^^16^^-^^18^^,^^20^^-^^22^^,^^25^^-^^28^ (Figure 2 and Table 2). According to the JBI, the level of evidence of the 17 studies was 1C.

**Table 2:** Risk of bias among included studies

Studies	Random sequence generation	Allocation concealment	Blinding of participants and personnel	Blinding of outcome assessment	Incomplete outcome data	Selective reporting of results
Chen *et al*. (2020)^13^	Low risk	High risk	Not informed	Not informed	Low risk	Low risk
De la Fuente *et al*. (2020)^14^	Low risk	High risk	High risk	Low risk	Low risk	Low risk
Presley *et al*. (2020)^15^	Low risk	Not informed	Not informed	Not informed	Low risk	Unclear risk^*^
White *et al*. (2020)^16^	Low risk	Not informed	Not informed	Not informed	Low risk	Low risk
Sharoni *et al*. (2018)^17^	High risk	Low risk	High risk	High risk	Low risk	Low risk
Santos *et al*. (2017)^18^	Low risk	Not informed	High risk	High risk	Low risk	Low risk
Paz-Pacheco *et al*. (2017)^19^	Low risk	Not informed	Not informed	Not informed	Low risk	Unclear risk^*^
Grillo *et al*. (2016)^20^	Low risk	High risk	High risk	Low risk	Low risk	Low risk
Pérez-Escamilla *et al*. (2015)^12^	Low risk	Not informed	Low risk	Low risk	Low risk	Low risk
Merakou *et al*. (2015)^21^	High risk	High risk	High risk	High risk	Low risk	Low risk
Ruggiero *et al*. (2014)^22^	Low risk	High risk	High risk	High risk	Low risk	Low risk
Plotnikoff *et al*. (2011)^23^	Low risk	Not informed	Not informed	Not informed	Low risk	Unclear risk^*^
Quinn *et al*. (2011)^24^	Low risk	Low risk	Not informed	Not informed	Low risk	Unclear risk^*^
Sönnichsen *et al*. (2010)^25^	Low risk	Low risk	High risk	High risk	Low risk	Low risk
Gaillard, *et al*. (2015)^26^	Low risk	Not informed	Not informed	Not informed	Low risk	Low risk
Gehlawat *et al*. (2019)^27^	Low risk	Not informed	Not informed	Not informed	Low risk	Low risk
Romero-Guevara *et al*. (2019)^28^	Low risk	Low risk	Low risk	Low risk	Low risk	Low risk

* Study registration or published protocol not found.

## Discussion

The results of the review made it possible to identify educational interventions in individuals with DM2 in primary health care, which were categorized into four main aspects, representing a challenge for nursing professionals seeking: control of the disease, adherence by the patient to the programs, adherence to the therapeutic regimen, showing to a positive impact on quality of life. Our results were consistent in showing that educational interventions have shown significant impacts on adherence and therapeutic satisfaction. 

This result agrees with other findings where the patient's adherence to the drug regimen showed a reduction in the severity of complications. It is believed that medication adherence factors in chronic patients can be made up of five major categories including economic and social factors, the health team, and the patient care system as well as treatment-related factors. Patient-related factors can be modified through education and increased knowledge.^29^Likewise, the evidence has shown the positive effects of educational interventions with an emphasis on self-care, these have shown improvements in self-efficacy during the health-disease processes faced by the population, highlighting an aspect that becomes relevant and that was evidenced in the results for coping with health conditions, such as empowerment and awareness of the disease. It is also shown that the inclusion approach of the patient and their family environment brings an improvement in knowledge and that it will thus have an influence on prevention of future complications such as foot care and other organs that may be affected.^4^^,^^30^ Through the application of these educational interventions, different strategies have been implemented, providing educational interventions individually and in groups. However, a meta-analysis supports our findings. It shows significant results to improve knowledge, self-control of the disease based on knowledge about the condition itself, and the treatment and identification of one's own abilities. This consequently brings about the reduction of HbA1C levels in self-care interventions aimed at groups (*p*<0.0001).^31^

Within this review, the relevant role of the use of strategies through technological resources was evidenced, giving an encouraging panorama in the combination of methodologies that seek to adapt to the specific conditions of the population and have shown a favorable impact on the lifestyle of patients presenting a reduction in HbA1C levels up to 0.38%. It also allows secondary results in the improvement of knowledge and other comorbidities, all this giving support for the combination of methodologies that will impact positive results both in the population and in the health system with the use of low-cost strategies.^32^ Therefore, showing the very positive results of educational interventions in patients with DM2, the nursing professional plays a very important role in the proper planning and execution of these patient-centered interventions for the self-control of the disease and its role in decision making, demonstrating with this relevant scope in the modification and obtaining of controlled clinical parameters in patients.^30^ Thus, educational interventions in patients with diabetes mellitus have identified a relevant reference point, when compared to care, not only because it involves compliance with figures between normal values in clinical parameters but also because it allows contributions in the implementation of these programs with different methodologies, multidisciplinary teams, and both individual and group approaches.^4^^,^^33^

Although this SR was carried out under PRISMA guidelines, it has some limitations. First, searches were only carried out in MEDLINE, EMBASE, LILACS, Web of Science and gray literature. Second, the analysis of the quality of the included studies showed lack of information on allocation concealment, blinding of outcomes assessment and blinding of participants and staff in some studies. Lastly, this review did not use the GRADE (Grading of Recommendations, Assessment, Development and Evaluation) methodology to evaluate the degrees of recommendation of the studies selected. Nonetheless, the JBI recommendations were used to assess the level of evidence of the studies.

## Conclusion

The findings of this review suggest that educational interventions in patients with DM2 in the primary health care setting can have a positive impact on therapeutic adherence, self-control and knowledge of the disease. In addition, it was possible to identify the influence of health teams, pointing out the scope of nursing professionals in the construction and implementation of educational interventions for better health outcomes. This way, the value of the performance of the nursing profession in its investigative, academic, practical, and management role that results in a contribution to the discipline and the community is pointed out.

## References

[1] Pan American Health Organization (2021). Enfermedades no transmisibles.

[2] Cho NH, Shaw JE, Karuranga S, Huang Y, da Rocha Fernandes JD, Ohlrogge AW (2018). IDF Diabetes Atlas: Global estimates of diabetes prevalence for 2017 and projections for 2045. Diabetes Res. Clin. Pract..

[3] He X, Li J, Wang B, Yao Q, Li L, Song R (2017). Diabetes self-management education reduces risk of all-cause mortality in type 2 diabetes patients: a systematic review and meta-analysis. Endocrine..

[4] Caro-Bautista J, Kaknani-Uttumchandani S, García-Mayor S, Villa-Estrada F, Morilla-Herrera JC, León-Campos Á (2020). Impact of self-care programmes in type 2 diabetes mellitus population in primary health care: Systematic review and meta-analysis. J. Clin. Nurs..

[5] Ferguson S, Swan M, Smaldone A (2015). Does Diabetes Self-management Education in Conjunction With Primary Care Improve Glycemic Control in Hispanic Patients?: A Systematic Review and Meta-analysis. Diabetes Educ..

[6] The Joanna Briggs Institute (2015). The Joanna Briggs Institute Reviewers’ Manual 2015: Methodology for JBI scoping reviews.

[7] Tricco AC, Lillie E, Zarin W, O’Brien KK, Colquhoun H, Levac D (2018). PRISMA extension for scoping reviews (PRISMA-ScR): Checklist and explanation. Ann. Intern. Med..

[8] Castillo-Merino Y, Ospina-Ayala C, Rodríguez-Acelas AL, Cañon-Montañez W (2021). Educational Interventions in Adults with Type 2 Diabetes Mellitus in Primary Health Care Settings: A Scoping Review Protocol. Inplasy Protoc..

[9] Higgins JPT, Altman DG, Gøtzsche PC, Jüni P, Moher D, Oxman AD (2011). The Cochrane Collaboration’s tool for assessing risk of bias in randomised trials. BMJ.

[10] Joanna Briggs Institute (2014). JBI Levels of Evidence FAME. JBI approach.

[11] McGuinness LA. “robvis: An R package and web application for visualising risk-of-bias assessments”.

[12] Pérez-Escamilla R, Damio G, Chhabra J, Fernandez ML, Segura-Pérez S, Vega-López S (2015). Impact of a community health workers-led structured program on blood glucose control among latinos with type 2 diabetes: the DIALBEST trial. Diabetes Care.

[13] Chen S, Qian D, Burström K, Burström B (2020). Impact of an educational intervention in primary care on fasting blood glucose levels and diabetes knowledge among patients with type 2 diabetes mellitus in rural China. Patient Educ. Couns..

[14] De la Fuente Coria MC, Cruz-Cobo C, Santi-Cano MJ (2020). Effectiveness of a primary care nurse delivered educational intervention for patients with type 2 diabetes mellitus in promoting metabolic control and compliance with long-term therapeutic targets: Randomised controlled trial. Int. J. Nurs. Stud..

[15] Presley C, Agne A, Shelton T, Oster R, Cherrington A (2020). Mobile-Enhanced Peer Support for African Americans with Type 2 Diabetes: a Randomized Controlled Trial. J. Gen. Intern. Med..

[16] White RO, Chakkalakal RJ, Wallston KA, Wolff K, Gregory B, Davis D (2020). The Partnership to Improve Diabetes Education Trial: a Cluster Randomized Trial Addressing Health Communication in Diabetes Care. J. Gen. Intern. Med..

[17] Sharoni SKA, Rahman HA, Minhat HS, Shariff-Ghazali S, Ong MHA (2018). The effects of self-efficacy enhancing program on foot self-care behaviour of older adults with diabetes: A randomised controlled trial in elderly care facility, Peninsular Malaysia. PLoS One..

[18] Santos JC, Cortez DN, Macedo MML, Reis EA, Reis IA, Torres HC (2017). Comparison of education group strategies and home visits in type 2 diabetes mellitus: clinical trial. Rev. Latino-Am. Enfermagem..

[19] Paz-Pacheco E, Sandoval MA, Ardena GJ, Paterno E, Juban N, Lantion-Ang FL (2017). Effectiveness of a community-based diabetes self-management education (DSME) program in a rural agricultural setting. Prim. Health Care Res. Dev..

[20] Grillo M de FF, Neumann CR, Scain SF, Rozeno RF, Beloli L, Perinetto T (2016). Diabetes education in primary care: a randomized clinical trial. Cad. Saude Publica..

[21] Merakou K, Knithaki A, Karageorgos G, Theodoridis D, Barbouni A (2015). Group patient education: effectiveness of a brief intervention in people with type 2 diabetes mellitus in primary health care in Greece: a clinically controlled trial. Health Educ. Res..

[22] Ruggiero L, Riley BB, Hernandez R, Quinn LT, Gerber BS, Castillo A (2014). Medical assistant coaching to support diabetes self-care among low-income racial/ethnic minority populations: randomized controlled trial. West. J. Nurs. Res..

[23] Plotnikoff RC, Eves N, Jung M, Sigal RJ, Padwal R, Karunamuni N (2010). Multicomponent, home-based resistance training for obese adults with type 2 diabetes: a randomized controlled trial. Int. J. Obes..

[24] Quinn CC, Shardell MD, Terrin ML, Barr EA, Ballew SH, Gruber-Baldini AL (2011). Cluster-randomized trial of a mobile phone personalized behavioral intervention for blood glucose control. Diabetes Care.

[25] Sönnichsen AC, Winkler H, Flamm M, Panisch S, Kowatsch P, Klima G (2010). The effectiveness of the Austrian disease management programme for type 2 diabetes: a cluster-randomised controlled trial. BMC Fam. Pract..

[26] Gaillard T, Amponsah G, Osei K (2015). Patient-Centered Community Diabetes Education Program Improves Glycemic Control in African-American Patients with Poorly Controlled Type 2 Diabetes: Importance of Point of Care Metabolic Measurements. J. Natl. Black Nurses Assoc..

[27] Gehlawat M, Lakshminarayanan S, Kar SS (2019). Structured Diabetes Education Program for Improving Self-care Behavior in Primary Care Settings of Puducherry: Evidence from a Randomized Controlled Trial. Indian J. Community Med..

[28] Romero Guevara SL, Parra DI, Rojas LZ (2019). Teaching: Individual” to increase adherence to therapeutic regimen in people with hypertension and type-2 diabetes: Protocol of the controlled clinical trial ENURSIN. BMC Nurs..

[29] Najafpour Z, Zeidi IM, Kalhor R (2021). The effect of educational intervention on medication adherence behaviour in patients with type 2 diabetes: Application of social marketing model. Clin. Diabetol..

[30] Coppola A, Sasso L, Bagnasco A, Giustina A, Gazzaruso C (2016). The role of patient education in the prevention and management of type 2 diabetes: an overview. Endocrine..

[31] Odgers-Jewell K, Ball LE, Kelly JT, Isenring EA, Reidlinger DP, Thomas R (2017). Effectiveness of group-based self-management education for individuals with Type 2 diabetes: a systematic review with meta-analyses and meta-regression. Diabet. Med..

[32] Haider R, Sudini L, Chow CK, Cheung NW (2019). Mobile phone text messaging in improving glycaemic control for patients with type 2 diabetes mellitus: A systematic review and meta-analysis. Diabetes Res. Clin. Pract..

[33] Choi TST, Davidson ZE, Walker KZ, Lee JH, Palermo C (2016). Diabetes education for Chinese adults with type 2 diabetes: A systematic review and meta-analysis of the effect on glycemic control. Diabetes Res. Clin. Pract..

